# Injectable Platelet-Rich Fibrin - A Revolution in Periodontal Regeneration

**DOI:** 10.7759/cureus.28647

**Published:** 2022-08-31

**Authors:** Monitha Gollapudi, Pavan Bajaj, Ranu R Oza

**Affiliations:** 1 Periodontology and Implantology, Sharad Pawar Dental College and Hospital, Datta Meghe Institute of Medical Sciences, Wardha, IND; 2 Periodontics and Implantology, Sharad Pawar Dental College and Hospital, Datta Meghe Institute of Medical Sciences, Wardha, IND

**Keywords:** platelet-rich fibrin, grafting, healing wounds, platelet concentrate, bone regeneration, growth factor, cell migration, platelet-rich plasma, platelets, collagen

## Abstract

As of a few years ago, platelet concentrates have been applied in a variety of medical and dental procedures. A notable aspect is that platelet-rich fibrin (PRF) is the most commonly utilized platelet concentrate in the field of dentistry. The most significant modification that was used over the years but had the biggest impact was injectable platelet-rich fibrin (I-PRF), which has more special properties. Additionally, the results of this I-PRF have been useful. The solid platelet-rich fibrin (PRF), which is a noticeable feature and has a low speed and duration in centrifugation, is the main advantage of I-PRF. I-PRF is primarily found in liquid form as PRF. It facilitates the quickening of increased vascularization and aids in accelerating the healing of wounds. An autologous blood concentration known as I-PRF has been known for many years.

The advantage of I-PRF is that it exhibits constant release of growth factors and promotes cell migration by announcing the expression of type I collagen and transforming growth factor mRNA. The majority of the time, plastic and orthopedic operations use injectable platelet aggregates. It also reduces adverse reactions to transplanted material as compared to other grafting techniques. Additionally, it makes numerous other operations, like regenerative ones, much better options. In circumstances where it has been noticed, I-PRF is helpful and crucial in periodontics for bone regeneration and wound healing. It is therefore not difficult to predict that this fully autologous blood concentrate, which is now being utilized in numerous applications and requires little invasiveness, will become even more frequently used in the future. This review paper contains the differences between platelet-rich plasma (PRP) and PRF, the development of diverse platelets, and the use of I-PRF in periodontal therapy.

## Introduction and background

Higher amounts of peptide growth factors can now be transported by platelet concentrates into periodontal lesions. Platelet-rich fibrin (PRF), a second-generation preparation included in the category of platelet-rich plasma (PRP) and first stated by Choukroun et al. in 2001, is the most effective platelet concentrate out of all those available [1]. First, in 1954, Kingsley introduced platelet concentrate, which later became known as platelet-rich plasma (PRP) [2]. It was created as a thrombocytopenia treatment. The initial attempt to use concentrated plate growth factors to aid in the healing of wounds during and after surgery involved several tries. Marx et al. first proposed the growth factor that is present in PRP and its concentration in their paper published in they discuss the results of platelet-rich preparation used in maxilla face reconstruction applications in articles from the year 1988 [3]. PRP preparation techniques vary according to protocols and take between 30 and 60 minutes to complete. In PRP, there are essentially two key centrifugation techniques used. Regarding the first centrifugation process, tubes coated with ethylene diamine tetra acetic acid (EDTA) and citric acid primarily serve to inhibit natural coagulation. In the second centrifugation step, bovine thrombin, calcium chloride, or any other artificial coagulant is introduced to the plasma to create artificial coagulation after the erythrocytes have been settled by the first centrifuge. It is renowned for its quick centrifugation technique as well [4]. First-generation platelet concentrate, or PRP as it is commonly known, can be in gel or liquid form. Following thrombin and calcium activation of centrifuged blood, it also manifests as a frail fibrin network. The issue is that we artificially included bovine thrombin and calcium chloride during the procedure; therefore, the product is not entirely autologous.

Ninety-five percent of the platelets in PRP come from the blood. These are the cells that directly influence other cells, such as osteoblasts, connective tissue cells, epithelium, and periodontal ligament cells. Even though platelet-rich plasma is crucial for delivering growth factors at various stages of wound healing, its major goal was to purge leukocytes from blood concentrates [5-6]. The two main drawbacks of platelet-rich plasma are that it is expensive and takes a long time to produce. The preparation process also involves a lot of steps. The second significant drawback is that the fibrin matrix structure created by artificial coagulation is stiffer than the fibrin matrix structure created by spontaneous coagulation [7].

Due to the drawbacks of first-generation PRP, platelet-rich fibrin, a second-generation blood product, was found. Since the discovery of platelet-rich plasma, platelet concentrates have been produced entirely autologously without the use of anticoagulants such as calcium chloride or artificial bovine thrombin throughout the production process. Simply put, it is created primarily by utilizing the patient's blood without the addition of any supplements. By manipulating the patient's blood, platelet-rich fibrin is essentially a surgical biological preservative; it also has a distinctive morphology. Additionally, it is crucial in preparing cells for tissue regeneration and is helpful in plastic surgery, maxillofacial surgery, and implant procedures. In the branch of periodontal therapy, PRF has many benefits in treating different types of periodontal defects [8-9]. PRF has another advantage: it has a complex three-dimensional fibrin framework. And when it comes to platelet rich fibrin (PRF) classification, it is done on the basis of centrifugal speed, time required for centrifugation and type of test tube used. Details of various types of PRF and their respective mode of preparation are given in Table 1.

**Table 1:** Types of PRFs PRF - platelet-rich fibrin

	Centrifugal speed	Centrifugal time	Tube type	Nature of the obtained PRF
Platelet rich fibrin (L-PRF), Choukroun 2000 [1]	2700 rpm	12 minutes	Glass tube	Solid
Titanium platelet-rich fibrin (T-PRF), Tunali 2014 [10]	2700 rpm	12 minutes	Titanium tube	Solid
Advanced platelet-rich fibrin (A-PRF), Choukroun 2014 [11]	1300 rpm	14 minutes	Glass tube	Solid
Albumin platelet-rich fibrin (Alb-PRF), Fujioka 2020 [12]	1300 rpm	8 minutes	Glass tube	Solid
Injectable platelet-rich fibrin (I-PRF), Mourao 2015 [13]	700 rpm	3 minutes	Plastic tube	liquid

Injectable platelet-rich fibrin (I-PRF) is the most recent and successful advancement in PRF. In essence, it was created by slowing down the liquid-based centrifugation approach and omitting the formation of a PRF membrane. I-PRF is referred to be an advanced type of PRF since it is injected (autologous PRF) into afflicted soft tissues, mucous membranes, or skin. It also has special qualities in the regeneration of human tissues. Gene therapy, tissue engineering, and platelets have all been demonstrated to be effective sites for signaling pathways such as platelet-derived factor growth. Biological intervention in regenerative therapies primarily comes in three forms. Injectable platelet-rich fibrin was created in 2001 by Choukroun et al. [1]. Leukocyte platelet-rich fibrin, a complex three-dimensional fibrin structure made primarily of 97 percent platelets and 50 percent leukocytes, was first created. Three layers are created after centrifugation: the uppermost layer is made up of platelet-poor plasma, the middle layer is made up of fibrin clots with a high concentration of platelets, and the lower layer is made up of red blood cells. But because these three coagulation layers formed without separation and the resorption duration is sufficient in soft healing, the desired outcome could not be achieved [10]. As a result, researchers began to design several types of PRF. To prevent the potential of silica particles hanging in the fibrin structure in a glass tube traveling to the patient, titanium platelet-rich fibrin (T-PRF) has been created. T-PRF has been seen to stay in tissue for longer than 30 days without causing any problems, and because of its lengthy resorption time and abundance of growth factors, this particular PRF was helpful in the repair of soft tissues [11]. Advanced platelet-rich fibrin (A-PRF) was created using a low centrifugation approach, which has resulted in a significant rise in the number of inflammatory cells and growth-promoting substances. As a result, in this instance, regenerative potential has grown [12-14]. 

In PRF, coagulation starts when blood and silica in a glass tube come into contact. T-PRF has a tighter fibrin network structure and contacts the titanium surface rather than silica when blood comes into touch with it. When compared to PRP, PRF's major drawback was that it was available in solid form. As a result, an I-PRF was produced; after a short time and low centrifugation speed, it was acquired in liquid form without building a PRF membrane [15-16]. 

Since injecting solid platelet-rich fibrin was not achievable, Miron et al. conducted fundamental research and discovered that liquid platelet-rich fibrin could be created by reducing the centrifugal speed and time duration below the specified levels. He claimed that a centrifugal speed of 60 g for three minutes allows for separation before clots have a chance to develop and also prepares the residual liquid in the state. Additionally, it was noted that only 1 to 1.5 ml of I-PRF were volumetrically present in a test tube containing 10 ml. The injectable platelet-rich fibrin can be injected into the skin or scalp of the face, and it remains a liquid for 10 to 15 minutes before it solidifies into a clot [17]. Additionally, PRF is crucial for the healing of wounds because it controls immunity, promotes angiogenesis, traps circulating stem cells, induces collagen synthesis, and stimulates the growth of fibroblasts and osteoblasts as well as the prolonged delivery of growth factors to the area where a wound is actually present.

## Review

Preparation of I-PRF

There are different types of preparation methods given by different researchers. Take a test tube, fill it with 9 to 10 ml of blood without adding any preservatives, and centrifuge it for two to three minutes at a speed of 3300 rpm to produce an orange-colored fluid that is thought to contain injectable platelet-rich fibrin, according to Mourao et al. [18]. Then, in 2009, AL-Malawi declared that, in accordance with the low-speed configuration approach, blood must first be collected in a test tube and immediately kept in a centrifuge at 600 rpm, 44g, for eight minutes. Following this procedure, a yellow i-PRF was created at an upper level, and other components were created or were already existent at a lower level [19]. 

Properties of I-PRF

The following are some of the characteristics of PRFs: i-PRF has demonstrated that it experiences increased cellular migration. Additionally, it was shown that PRP and i-PRF demonstrated comparable tissue compatibility. Additionally, we can see that i-PRF has a bone transplant bonding mechanism that helps with the right adaption of the defect area. Fibronectin, an extracellular glycoprotein, is the main component of injectable platelet-rich fibrin. Fibronectin has a big molecular weight. Additionally, applying fibronectin to the surfaces of roots promotes cellular growth. From supra-crestal components to periodontal ligaments, cellular growth spreads. Last but not least, I-PRF offers higher biologic qualities than PRP.

Merits and demerits of I-PRF

Merits 

It is simple to prepare and use, and there is no biological modification. Additionally, it facilitates cellular motility and cytokine enmeshing. The majority of medication is in injectable form, which also lessens potential consequences. Additionally, because more growth factors are produced, it has a greater ability to activate regenerative cells. In addition, it creates a tiny fibrin clot that allows it to function as a dynamic gel. Last but not least, and perhaps most crucially, it is a straightforward and affordable procedure regardless of one's financial situation. Additionally, it is crucial for the release of growth factors for 10 to 12 days.

Demerits

Because I-PRF is made in small amounts from autologous blood, it only applies to a small portion of general surgery. The primary clinical benefit of I-PRF is based on the short handling time between blood collection and centrifugation since platelet-rich plasma is created without the use of additional anticoagulants. Another significant drawback is that the fibrin matrix is only usable for that individual donor since it contains circulating immune cells and highly antigenic plasmatic chemicals. Additionally, if stored I-PRF is not used right away, it may get contaminated with germs.

Application in periodontal therapy

Mourao et al., in 2015, stated that platelet-rich plasma can be replaced by I-PRF when used with biomaterials in bone grafting as a platelet concentrate for bone regeneration [18]. Injectable platelet-rich fibrin has the ability and potential to release larger amounts of a variety of growth factors, according to research by Miron et al. [17]. Miron et al. from 2017. Additionally, it involves the expression of increased quantities of platelet-derived growth factor (PDGF), transforming growth factor (TGF), collagen 1, and fibroblast migration [17]. Chenchev et al. (2017) demonstrated through successful radiographic and clinical outcomes that combining advanced platelet-rich fibrin (A-PRF) with injectable platelet-rich fibrin (I-PRF) is beneficial for bone argumentation of the alveolar ridge prior to or during implant placement [20]. According to Wang et al. (2018), in control tissue culture, PRP promotes osteoblast migration by a factor of two, whereas i-PRF displays a factor of three, indicating that i-PRF exhibits stronger osteoblast differentiation and proliferation [21]. In accordance with Varela et al. (2018), I-PRF, which contains platelets, leukocytes, type 1 collagen, osteocalcin, and growth factors, is an excellent or extremely helpful option for the healing of soft and mineralized tissue [22]. According to Gode et al. 2019, I-PRF improved the postoperative survival rate of diced cartilage [23]. According to Izol et al. I-PRF has a favorable impact on root coverage in free gingival graft surgery [24]. Ozsagir et al. 2020 found that for people with thin phenotypic, combining injectable platelet-rich fibrin with micro-needling had the greatest potential to increase gingival thickness. The results also revealed that the first step in non-surgical approaches for enhancing and improving gingival thickness can be thought of as a combination of injectable platelet-rich fibrin and micro-needling [25]. Turer et al., in 2020, stated that gingival recession decreases more in group applied I-PRF in operations with coronally advanced flap with a connective tissue graft [26].

Adding I-PRF to a coronally advanced flap and combining it with a connective tissue graft resulted in the development of increasing keratinized tissue height and decreasing recessive depth when compared to combining the coronally advanced flap (CAP) only with a connective tissue graft, according to research by Turer et al. in 2020 [26]. Combining advanced injectable platelet-rich fibrin and injectable platelet-rich fibrin appears to improve bone formation in alveolar clefts while reducing bone resorption and increasing bone volume, according to Dayashankara Rao et al. in 2021 [27]. He added that secondary alveolar grafting, if necessary, improves or boosts periodontal health.

Uses in various fields

I-PRF is used as an injection for a variety of conditions, including osteoarthritis, meniscus healing, alopecia, sports injuries, tendon/ligament injuries, musculoskeletal regenerative producers, and acne. It is also used in areas such as facelift surgery, knee arthroplasty, and heart surgery to reduce the incidence of infections. 

## Conclusions

Clinicians and researchers in the field of dentistry need to conduct much more research in tissue transmission engineering to fully understand platelet concentration's benefits and applications in various fields. It has beneficial effects to allay worries about the disease and immunogenic reactions because it is an entirely natural, physiological, and affordable source of an autologous product. There is more to learn about platelet concentration, and more work needs to be done on tissue transmission. And in this regard, injectable platelet-rich fibrin-which introduced the usefulness and functionality of the application of platelet concentrates-was the cleverest development in the field of platelet-rich fibrin. Additionally, it affects osteoblastic behavior, which aids in the significant release of growth factors when combined with a variety of biomaterials. Therefore, the presence of platelets and growth factors can convert an osteoconductive graft into an osteopromotive one. 
